# Chronic stress is associated with reward and emotion-related eating behaviors in college students

**DOI:** 10.3389/fnut.2022.1025953

**Published:** 2023-01-12

**Authors:** Muyesaier Tuluhong, Pengfei Han

**Affiliations:** ^1^Faulty of Psychology, Southwest University, Chongqing, China; ^2^MOE Key Laboratory of Cognition and Personality, Chongqing, China

**Keywords:** chronic stress, food odor perception, emotion-related eating, reward-related eating, food preference

## Abstract

**Introduction:**

Stress is related to altered olfactory perception and eating behaviors. The current study investigated the association between chronic stress, food reward and perception of food and non-food odors among college students.

**Methods:**

Sixty-one participants completed the Perceived Stress Scale (PSS) and the Brief Daily Stressors Screening Tool (BDSST). The detective threshold and suprathreshold perception (pleasantness, intensity, and familiarity) of two food (chocolate, strawberry) odors and a non-food (rose) odor were measured. Food reward and macronutrient preference were measured using the computerized Leeds Food Preference Questionnaire and the Macronutrient and Taste Preference Ranking task, respectively. Reward-related eating, emotional eating and eating-related inhibitory control were measured by the Dutch Eating Behavior Questionnaire (DEBQ) and the Reward-Based Eating Drive Scale (RED) scales.

**Results:**

Neither the perceived stress or the severity of daily life stressor exposure was related to odor sensitivity, however, the PSS score was significantly correlated with pleasantness for strawberry odor (*r* = 0.329, *p* = 0.013). Chronic stress (PSS and BDSST scores) was significantly correlated with the DEBQ emotional eating and reward-related eating measured by RED (all *ps* < 0.01). Moreover, the BDSST score was negatively correlated with subjective liking for low-calorie sweet foods (*r* = −0.46, *p* < 0.001).

**Discussion:**

Together, our preliminary results suggest disassociated effect of chronic stress on odor perception and eating behaviors.

## Introduction

Olfaction plays an important role in eating. Food odors are potent stimuli with high ecological relevance and more affect-laden (e.g., hedonism or motivation) in the context of food appraisal (1). Chronic stress is characterized as an oppressive, unremitting prolonged aversive state that accumulate and lead to poor psychological and physical health (2). Exposure to chronic stress is associated with long-lasting effects on olfactory functions (3, 4). Specifically, mice exposed to chronic variable stress demonstrated decreased sensitivity toward food (lemon and strawberry) and non-food odors (5, 6), as well as reduced odor-induced electrophysiological responses at the olfactory mucosa (7). In humans, chronic stress exposure is associated with structural and functional alterations of several key brain areas that involved in olfactory processing, such as the amygdala and the hippocampus (8). However, the potential relationship between chronic stress levels and odor perceptions in humans had not been directly explored. Besides, food odors can reflect nutritional information such as the caloric density and main macronutrients content of food (9). For example, by smelling of food odors, people can distinguish and can classify food items with the “taste” (for example: sweet, non-sweet) or energy density (e.g., high or low energy-dense) (10, 11). It is also relevant to investigate the relationship between chronic stress with food odors related to high or low-calorie density.

With large cohort of population survey, several recent studies have shown an association between high chronic stress levels and unhealthy dietary patterns, such as increased consumption of foods with high fat or sugar contents, and decreased preference and consumption of fruits or vegetables (12–14). One possible mechanism is that stress increases the reward processing of food *via* cortisol activation (15) and reduced dietary restraint (16). For example, people with high chronic stress demonstrated enhanced brain responses to high-calorie food cues (17). In addition, chronic stress was positively correlated with other abnormal eating behavior, such as emotional eating (18), loss of control of eating (19).

The aim of the current study was twofold. First, the association between chronic stress and perception of food (chocolate and strawberry) and non-food (rose-like) odors were investigated. Second, food reward and macronutrient preferences were measured with validated behavioral tests, and the relationship between chronic stress with food reward and preference were explored. Moreover, the stress responses (subjective perceived stress) and stressor exposure of participants were assessed using the perceived stress scale (PSS) (20) and the brief daily stressors screening tool (BDSST) (21).

## Materials and methods

### Participants

Participants were recruited from college students of the Southwest University China. Intended participants with known olfactory dysfunction, stuffy nose or rhinitis, neurological or mental diseases, smoking habit, or taking drugs that affecting appetite or olfaction were excluded *via* online survey. Six-one college students (Age Mean = 20.7, SD = 2.0; BMI Mean = 20.0, SD = 2.2; 33 females) were recruited and participated in the study. All participants reported to have normal olfactory function, and none was tested to be COVID-19 positive. The experiment was carried out in accordance to the Declaration of Helsinki on biomedical research involving human subjects. The protocol was approved by the Ethics Committee at the Faculty of Psychology Southwest University. Participants signed consent form prior to participation.

### Procedure

Participants were instructed not to wear perfumes and to avoid eating anything (water excepted) 2 h before they come to the laboratory. Upon arrival, participants rated their hunger level on a 100-mm visual analog scale (from 0 = not hungry at all to 100 = very hungry). Participants were asked to fill out the questionnaires, then olfactory tests in the order of evaluation task, threshold test and discrimination tasks were performed. After olfactory tests, participants performed the food reward and macronutrients preference tasks. The whole experiment lasts for about 1 h.

### Questionnaires

#### Perceived stress and daily stressor

The Chinese version of the PSS (22) was used to measure participants’ long-term stress level. The 10-item scale is used to assess how out of control, unpredictable or overloaded an individual’s life has felt in the past month on a five-point scale (0 = never, 4 = always) (20). The ratings of all items were summed to create a score range from 0 to 40, with higher score indicating a higher level of chronic stress. The PSS offers a good internal reliability (Cronbach’s α = 0.88).

The BDSST is a 10-item questionnaire assessing the experience of general daily stressors in eight distinct life domains such as housing or employment or study over the past 12 months (21). It measures subjective degree of stress on a five-point scale (0 = not at all, 4 = very much). Due to the cultural difference, the first item “difficulty in social obligation” was deleted, so the questionnaire had nine items in total. The internal reliability of the Chinese version of the BDSST was high, and the Cronbach’s α coefficient was 0.78.

#### Chronic stress related manifestation

As suggested by Schmidt et al. (23), chronic stress is characterized as a multidimensional including fatigue, depressiveness and anxiety. Relevant questionnaires were included to assess those aspects. The Chalder Fatigue Scale (CFS) measures participants’ physical and mental fatigue in the past month (24). The CFS consists of 11 items with each question is rated on a four-point scale (0 = less than usual, 1 = no more than usual, 2 = more than usual, 3 = much more than usual). The higher the score of each dimension, the higher the fatigue degree. The internal reliability of CFS was also good (Cronbach’s α = 0.81). The Chinese version (25) of Beck Depression Inventory (BDI) was used to measure participants’ depressive tendencies (26). The scale consists of 13 questions, each of which has 4 short sentences, representing 4 possible answers. The Chinese version of the BDI offers a good internal reliability with a Cronbach’s α coefficient of 0.88. The Chinese version (27) of Trait Anxiety Inventory (STAI-T) was used to assess subjects’ relatively stable anxiety tendencies, including the general states of calmness, confidence and security (28). The scale consists of 20 items, and each item is rated on a four-point scale (1 = almost none, 2 = some, 3 = often, 4 = almost always). The higher the total score, the higher the trait anxiety level. The internal reliability of the Trait Anxiety Inventory was 0.89.

#### Eating behaviors

The Dutch Eating Behavior Questionnaire (DEBQ) was used to measure individuals’ problematic eating tendencies (29). The questionnaire consists of 33 questions and measures three dimensions: restrained eating, emotional eating, and external eating. Each question is rated on a five-point scale (1 = never, 2 = occasionally, 3 = sometimes, 4 = always, 5 = very often). The higher the score of each dimension of the questionnaire is, the higher the tendency of restricted eating, emotional eating and external eating. The internal reliability of the three subscales was high (Cronbach’s α ranged from 0.73 to 0.91).

The 13-item reward-based Eating Drive Scale (RED-13) measures reward-related eating (30). The scale assesses three dimensions: loss of control over eating, lack of satiety, and pre-occupation with food. Participants answered each question on a five-point scale from 1 (strongly disagree) to 5 (strongly agree). The higher the score of each dimension of the questionnaire, the higher the tendency to overeat. The internal reliability of the three subscales was good (Cronbach’s α ranged from 0.77 to 0.88).

#### Olfactory measurement

A high-calorie food odor (chocolate, Taste Master Pty Ltd, Australia, Product code ITM40033), a low-calorie food odor (strawberry, Taste Master Pty Ltd, Australia, Product code ITM20177) and a non-food odor (rose, Taste Master Pty Ltd, Australia, Product code IFP10765/B) were selected as olfactory stimuli. The edibility rating of the rose odor was significantly lower than that of the chocolate or the strawberry odors (*p* < 0.001).

Participants first evaluated the three odors at suprathreshold concentrations (chocolate 0.128%; strawberry 0.128% and rose 25.6%). Participants held the brown bottle and sniffed it for three to five seconds and rated for the odor pleasantness, intensity, or familiarity on 100-mm visual analogue scales from 0 (very unpleasant/no smell at all/very unfamiliar) to 100 (very unpleasant/very intense/very familiar). After completion of the suprathreshold odor evaluation tests, participants underwent the odor sensitivity test. Odors were diluted using propylene glycol into 12 concentrations in a geometric dilution series (1:2). The assessment of participants’ olfactory sensitivity followed the single-staircase, 3-alternative forced choice procedure, in which participants were presented with 3 randomly arranged bottles, 2 of which contained pure diluent (the propylene glycol) and the third the target odor stimuli. Participants have to decide which smells differently. The test follows the procedure of the “Sniffin’ Sticks” odor threshold test (31). In brief, two successive correct identifications or one incorrect identification triggered a reversal of the staircase, i.e., the next higher or the next lower concentration step was presented, respectively. Seven reversals had to be obtained (including the starting point), and the sensitivity was defined as the mean of the last four staircase reversals. A higher score indicates higher odor sensitivity. The order of olfactory test for three odors was balanced among participants.

After completion of the odor threshold tests, participants evaluated the three odors at suprathreshold concentrations (chocolate 0.128%; strawberry 0.128% and rose 25.6%). Participants held the brown bottle and sniffed it for three to 5 s and rated for the odor pleasantness, intensity, or familiarity on 100-mm visual analog scales from 0 (very unpleasant/no smell at all/very unfamiliar) to 100 (very unpleasant/very intense/very familiar).

#### Food reward

Food liking and wanting were measured by the computer-based Leeds Food Preference Questionnaire (32). Twenty food pictures were selected based on their taste (sweet or savory) and fat content (high or low) resulted in four categories (high-fat sweet (HFSW); high-fat savory (HFSA); low-fat sweet (LFSW), and low-fat savory (LFSA)] with five foods in each category (Supplementary Table 1). The food pictures were selected based on International Food Picture Database and modified for local Chinese dietary culture.

The task consisted of two parts, presented in randomized order. For explicit liking, participants rated for each food item on a 100-mm visual analogue scale (VAS). For wanting part, there are 190 groups of 2 food pictures in each group for the current desired choice. Two food pictures in each group were presented on the left and right positions of the same page in a random order. Subjects were required to press “c” and “m” keys on the keyboard for their key response. Finally, the cumulative bonus points of the degree of wanting of each food picture are accumulated to calculate the degree of food wanting of each food.

#### Macronutrients and taste preference

The Macronutrient and Taste preference ranking task (MTPRT) was developed for measuring individuals’ preference for food tastes and macronutrients (33). A modified version (10) of the MTPRT includes 32 food images from four macronutrient categories: high-carbohydrate, high-fat, high-protein and low-energy. Each category contained 8 food items, of which four were sweet and four were savory. Because no products met all requirements to be included as high-protein sweet, the high-protein category formed an exception and consisted of 8 savory products. Therefore, the seven categories are as follows: high-carbohydrate sweet (HCSW), high-carbohydrate savory (HCSA); high-fat sweet (HFSW); high-fat savory (HFSA); high-protein savory (HPSA); low-energy sweet (LESW); low-energy savory (LESA) and savory (LESA) (Supplementary Table 2). There were three parts in the test: practicing, liking, and ranking. The liking part was designed to introduce participants to each product by name and picture. Liking was assessed by presenting pictures of all 32 food items with the question: “*How much do you like [product name]*?” which was rated on a 9-point scale ranging from 1 (do not like at all) to 9 (like extremely). The ranking part consisted of two sections, one focused on macronutrients and the other on taste, i.e., sweet and savory. Participants were asked to rank four products based on how much they preferred to eat the different foods in their daily life. The task outcomes were: macronutrient liking score (range from 1 to 9), macronutrient preference score (range from 1 to 4), and taste preference score (range from 1.5 to 3.5). These rankings were used to assess the relative preferences for specific macronutrient or flavors (sweet or savory). The task was executed in E-Prime 2.0 professional (Psychology Software Tools, Pittsburgh, PA, USA).

#### Data analysis

First, we checked whether the variables for approximately normally distribution, by use of Shapiro–Wilk’s tests. The gender, age, BMI and hunger level of the subjects was included as control variables for the following analysis. However, PSS and BDSST were used as predictor variables to investigate the associations between stress and odor perception. Correlation analyses were performed between the chronic stress measures with olfactory measures, eating behaviors and food reward. Notably, the significance was established for α = 0.05, and the obtained *p*-values were corrected for multiple comparisons (e.g., α = 0.017 = 0.05/3 for odor sensitivity). For odor pleasantness, odor familiarity was included as control variables, as these two are positively linked (34). All analyses were performed by means of the SPSS (version 26.0, SPSS Inc., Chicago, IL, USA) and GraphPad Prism 8 (GraphPad Software, Inc. La Jolla, CA, USA) software. Significance level was set at *p* < 0.05.

## Results

### Participant characteristics

Table 1 illustrated the descriptive statistics of major variables. The high level of perceived stress is characterized by higher level of fatigue, depressive symptoms, and trait anxiety. As suggested the perceived stress is typically multifaceted and includes several psychological components of the stress response, such as feeling of fatigue or anxiety (2, 23). The following analyses will focus on the two variables that assess chronic stress.

**TABLE 1:** Correlations, means, standard deviations of scale variables (*N* = 61).

	1	2	3	4	5
1. PSS	-				
2. BDSST	0.62***	-			
3. STAI-T	0.74***	0.54***	-		
4. BDI	0.56***	0.43**	0.63***	-	
5. CFS	0.34**	0.35**	0.36**	0.60***	-
Mean	15.46	11.77	43.08	4.41	14.30
Standard deviation	4.86	4.81	8.08	4.57	4.62
Range	6–27	2–25	29–64	0–17	1–27

**p < 0.01; ***p < 0.001. PSS, perceived stress scale; BDSST, brief daily stressors screening tool; CFS, chalder fatigue scale; BDI, beck depression inventory; STAI-T, trait anxiety inventory.

### Odor perception

A weak significant correlation was observed between the BDSST and threshold for rose odor (*r* = 0.270, *p* = 0.038). However, the correlation no longer significant after corrected for multiple tests (*p* > 0.017). No significant correlation was found between the PSS, or the BDSST and odor threshold for chocolate or strawberry odors. There was neither significant correlation between stress measures and odor discrimination ability.

Regarding the supra-threshold odor perception, there was no significant correlation between PSS or BDSST and pleasantness or intensity ratings for chocolate or rose odors, after controlling for odor familiarity. For the strawberry odor, PSS was significantly correlated with pleasantness ratings (*r* = 0.312, *p* = 0.017), but not for intensity ratings (*r* = −0.08, *p* = 0.57). The significance level was preserved after odor familiarity and intensity ratings were both controlled (*r* = 0.329, *p* = 0.013). In other words, a higher perceived stress is related with higher pleasantness for strawberry odor.

### Questionnaire-based eating behavior

**FIGURE 1:**
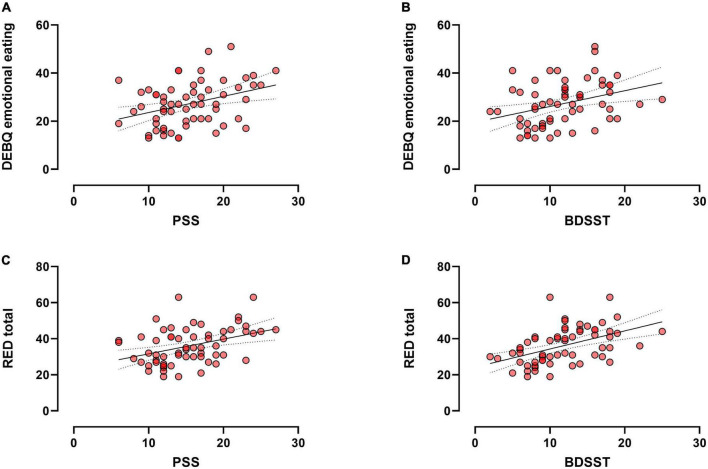
Scatter plot (*N* = 61) showing significant correlations between the DEBQ emotional and PSS **(A)** or BDSST **(B)**; and the significant correlations between the RED total score and PSS **(C)** or BDSST **(D)**.

The DEBQ emotional eating was significantly correlated to the PSS (*r* = 0.327, *p* = 0.012; Figure 1A) and BDSST (*r* = 0.352, *p* = 0.006; Figure 1B). No significant correlation was found between DEBQ restraint or external eating scores and PSS or BDSST (*ps* > 0.1). Regarding reward-related eating, the RED total score was significantly correlated to PSS (*r* = 0.39, *p* = 0.002; Figure 1C) and BDSST (*r* = 0.48, *p* < 0.001; Figure 1D). Specifically, the PSS was significantly correlated to the loss of control eating (*r* = 0.350, *p* = 0.007) and the lack of satiety (*r* = 0.392, *p* = 0.002) of the RED scale, but not the food preoccupation (*r* = 0.19, *p* > 0.1). The BDSST score was significantly correlated to all the subscales of the RED scale, namely loss of control (*r* = 0.447, *p* < 0.001), food preoccupation (*r* = 0.427, *p* = 0.001), and the lack of satiety (*r* = 0.487, *p* < 0.001).

### Food liking, wanting, and macronutrients preference

**FIGURE 2:**
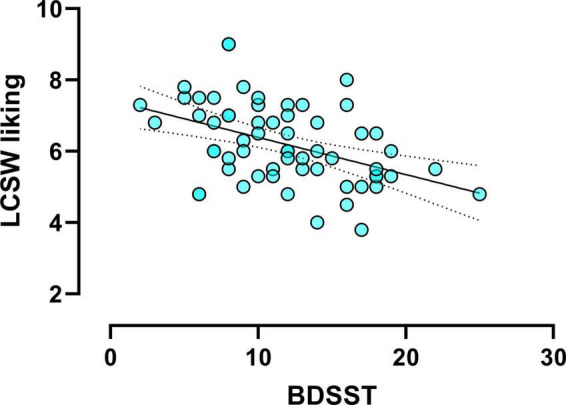
Scatter plot (*N* = 61) showing the significant correlation between daily exposed stressors [measured using the brief daily stressors screening tool (BDSST)] and the liking ratings for low-calorie sweet (LCSW) foods (*r* = –0.46, *p* < 0.001).

There was a negative correlation between the BDSST and liking for low-calorie sweet food (*r* = −0.46, *p* < 0.001, Figure 2). That is to say, the higher BDSST score, the lower liking for low-calorie sweet foods. No other significant correlation was found between chronic stress and food evaluation task measures.

## Discussion

Animal research showed that chronic stress is related to olfactory impairment, especially early step of olfactory processing, such as odor detection (6, 7). However, our primary analyses indicated no significant association between chronic stress and olfactory sensitivity to chocolate, strawberry or rose odors. The olfactory habituation/cross-habituation test was used in the animal study which is different from the detection threshold test used for human study. In addition, the well-controlled chronic stress induction in animal experiments led to severe depressive like status. Thus, direct comparison of the current results with findings from animal studies can be difficult. Emotions have been linked to changed odor perception. Chronic stress can induce mood changes and promote the onset of anxio-depressive symptoms in human with loss of pleasure seeking (anhedonia) (35). For examples, associations between state anxiety and decreased sensitivity to rose odor (36), while another study found people with high anxiety sensitivity demonstrated enhanced sensitivity to guaiacol–a smoke-like odor (37). Future study would include measures of chronic stress associated negative emotions of the participants and investigate the effect of specific emotional feelings on odor perception (3). There were evidences from animal research that stress-related decreased olfactory perception is modulated by the glucocorticoid hormone (5, 38). In contrary, early human research showed that increased cortisol was associated with improved odor detection abilities (39). Future research with cortisol measurement included could better characterize the impact of chronic stress on olfaction.

In terms of eating behavior, participants with a higher level of perceived stress demonstrated higher tendency for reward-based eating, with higher score on loss of control eating and lack of satiety, which is in accordance with previous research findings (40–42). It had been shown that participants with higher reported chronic stress demonstrated increased activation of the reward brain regions and decreased activations of the prefrontal regions in response to high palatable food stimuli (17). However, the current study did not show significant results regarding the food reward processing assessed with the food reward and preference tasks. One study has found that the level of chronic stress during examination period had no effect on behaviors related to palatable food purchase and intake, but participants reported having less control over their food choices during the exam period (43). Moreover, a significant association was found between the BDSST and decreased liking for low-calorie foods. This is in consistent with previous study showing a decreased hedonic ratings and liking for low-calorie chip flavors among individuals with high chronic stress levels (44). However, the results also showed positively correlation between the perceived stress score and pleasantness rating for the strawberry odor at suprathreshold level.

There are limitations to the current study. First, limited types of odor were used in the olfactory perception tests, which is insufficient to draw any generalized conclusions. Second, compared to other studies using PSS, the participants within the higher range of PSS score may be moderate stress. Other potential moderating factors (e.g., gender) was not explored with the small sample size in the current study. In addition, the computerized tasks may be not optimal do not capture the real liking perception of food flavors (45). Last but not least, some questionnaires (e.g., RED) lack of local validation. The results from the current study could not be generalized to wider population (e.g., different ages, or geographical backgrounds).

In conclusion, the current preliminary study provided little evidence for a correlation between chronic stress levels and odor perception. Higher chronic stress was associated with reward-related and emotional eating behaviors and decreased liking for low-calorie sweet foods. Future research on characterizing stress-related eating favor a multidimensional and more objective measurement of chronic stress and also a combination of self-reported eating behavior with actual food choices and consumption (46, 47).

## Data availability statement

The original contributions presented in this study are included in the article/Supplementary material, further inquiries can be directed to the corresponding author.

## Ethics statement

The studies involving human participants were reviewed and approved by the Ethics Committee at the Faculty of Psychology Southwest University. The patients/participants provided their written informed consent to participate in this study.

## Author contributions

MT and PH contributed to conception and design of the study. MT performed the experiment and the statistical analysis and wrote the first draft of the manuscript. PH guided the conduct of experiments, data analysis, and revision of the manuscript. Both authors contributed to manuscript revision, read, and approved the submitted version.
