# Supplementary material for: Chronic stress is associated with reward and emotion-related eating behaviors in college students
Source: Front Nutr. 2023 Jan 12;9:1025953. doi: 10.3389/fnut.2022.1025953 (PMC9879612; doi:10.3389/fnut.2022.1025953)
Supplement: Supplementary file 1 [file Table_1.DOCX]

**Supplementary materials**

| **Table 1** Description and categories of photographic food stimuli in Food Preference test | | | | |
| --- | --- | --- | --- | --- |
| **HFSA** | **LFSA** | **HFSW** | **LFSW** |  |
| Roast chicken wing | Snack mix | Moon cakes | Gummi candy |  |
| Pistachios | Spaghetti | Fruit cake | Strawberries |  |
| Chips | Spiced corned egg | Filled chocolates | Fruits |  |
| Peking duck | Shrimp | Matcha tiramisu | Peaches |  |
| Roast sausage | Potato wedges | Shortbread cookies | Banana |  |
| HFSA: high-fat savory; LFSA: low-fat savory; HFSW: high-fat sweet; LFSW: low-fat sweet | | | |  |

| **Table 2** Description and categories of photographic food stimuli in Macronutrient and Taste Preference Ranking task | | | | | | |
| --- | --- | --- | --- | --- | --- | --- |
| **HCSW** | **HCSA** | **HFSW** | **HFSA** | **HPSA** | **LESW** | **LESA** |
| Moon cakes | Snack mix | Macaron | Peking duck | Roasted chicken | Strawberries | Salad |
| Fruit cake | Spaghetti | Filled chocolates | Pistachio | Salmon | Apple | Tomatoes |
| Filled waffles | Fried prawn crackers | Brownie with nuts | Chips | Spiced corned egg | Banana | Mixed vegetables |
| Gummi candy | Rice waffles | Matcha Tiramisu | Walnut | Ham cut | Peaches | Mixed salad |
|  |  |  |  | Steak |  |  |
|  |  |  |  | Sushi rolls |  |  |
|  |  |  |  | Shrimp |  |  |
|  |  |  |  | Fried chicken cutlet |  |  |
| HCSW: high-carbohydrate sweet; HCSA: high-carbohydrate savory; HFSW: high-fat sweet; HFSA: high-fat savory; HPSA: high- protein savory; LESW: low- energy sweet; LESA: low- energy savory | | | | | | |
